# Effects of Noisy Galvanic Vestibular Stimulation on the Muscle Activity and Joint Movements in Different Standing Postures Conditions

**DOI:** 10.3389/fnhum.2022.891669

**Published:** 2022-06-02

**Authors:** Tsubasa Mitsutake, Takanori Taniguchi, Hisato Nakazono, Hisayoshi Yoshizuka, Maiko Sakamoto

**Affiliations:** ^1^Department of Physical Therapy, Faculty of Medical Science, Fukuoka International University of Health and Welfare, Fukuoka, Japan; ^2^Department of Occupational Therapy, Faculty of Medical Science, Fukuoka International University of Health and Welfare, Fukuoka, Japan; ^3^Faculty of Medicine, Education and Research Center for Community Medicine, Saga University, Saga, Japan

**Keywords:** standing posture, noisy galvanic vestibular stimulation, lower limb, angular velocity, joint movement, muscle activity

## Abstract

**Objective:**

Noisy galvanic vestibular stimulation (nGVS) is an effective method for stabilizing posture; however, little is known regarding the detailed muscle activity and joint movement in the standing posture. This study aimed to clarify the changes in the lower limb muscle activity and joint angular velocity by nGVS intervention using the simultaneous assessment method of inertial measurement units and surface electromyography (EMG).

**Methods:**

Seventeen healthy participants were assessed for their physical responses under four conditions (standing on a firm surface with eyes-open/eyes-closed, and a foam surface with eyes-open/eyes-closed) without stimulation (baseline) and with stimulation (sham or nGVS). Noise stimuli were applied for 30 s at a level below the perceptual threshold. The body control response was evaluated using EMG activity and angular velocity of the lower limbs.

**Result:**

Regarding the change from baseline for each parameter, there was a significant interactive effect of EMG activity in the muscle type × intervention and EMG activity and angular velocity in the condition × intervention. *Post hoc* analysis revealed that the angular velocity was significantly decreased in the abduction-adduction direction in the standing on a foam surface with eyes-closed condition compared to that with eyes-open in the nGVS intervention.

**Conclusion:**

Our results suggest that nGVS altered physical responses in different standing postural conditions. The present study is exploratory and therefore the evidence should be investigated in future studies specifically target those muscle activities and joint motion parameters.

## Introduction

The ability to maintain a stable standing posture is related to various body control functions. Considering the postural control systems associated with balance ability, sensory strategies contribute to postural stability by facilitating the interaction of the visual, somatosensory, and vestibular senses with the environmental and individual posture changes (Peterka, 2002; Horak, 2006). In particular, the vestibular sensory system provides information regarding the position and movement of the head with respect to gravity and inertial forces. Rehabilitation focusing on vestibular sensory function can improve the balance ability of the elderly (Rossi-Izquierdo et al., 2020) as well as patients with neurological diseases (Mitsutake et al., 2020; Tramontano et al., 2021) and vestibular disorders (Hall et al., 2021). Thus, an effective vestibular intervention could prevent falls.

In the novel vestibular intervention method, noisy galvanic vestibular stimulation (nGVS) activates the vestibular cortex by applying a weak noise current to the vestibular end organs and their afferents *via* electrodes placed bilaterally over the mastoid process (Mitsutake et al., 2021; Valdés et al., 2021). This stimulation may modulate the threshold or excitability of the motor response by vestibular input through stochastic resonance of noise addition to non-linear systems, inducing a change in the plasticity of information processing in the neural systems (McDonnell and Ward, 2011). In addition, subthreshold sinusoidal and stochastic noise can modulate the sensitivity of individual neurons in the medial vestibular nucleus without affecting the basal firing rates (Stefani et al., 2019). In contrast to the conventional galvanic vestibular stimulation using direct current, nGVS provides stimulation for enhancing sensory inputs in the vestibular afferents without directional specificity (Ko et al., 2020). Considering that input information from the vestibular sensory system affects the postural control system, nGVS could possibly result in improvements in the postural control function, thereby serving as a beneficial intervention focusing on the vestibular system.

Postural control studies using nGVS have reported that stimulus intervention improves body balance in adults regardless of the age or type of vestibular disorders (Goel et al., 2015; Wuehr et al., 2017; Fujimoto et al., 2018). Other previous studies demonstrated that the center of pressure sway decreased in young and community-dwelling elderly individuals who maintained a standing position under various conditions visually (open vs. closed eyes) and on the floor (firm vs. foam) during nGVS (Iwasaki et al., 2014; Inukai et al., 2018a,b). nGVS may effectively lower the vestibular threshold for eliciting balance-related reflexes necessary for adequate regulation of postural equilibrium (Schniepp et al., 2018). Among the various standing postural conditions, closed-eye standing on an unstable surface controls posture through a vestibular-dominant sensory strategy (Horak, 2006; Shumway-Cook and Woollacott, 2012). However, it is unclear whether muscle activity and joint movements are affected by different standing conditions. The standing posture control system contributes to the postural stability; not only considering the sensory strategies, including the vestibular system, but also the physical responses of movement strategies using ankle and hip joint movements (Horak, 2006). Since the standing stabilization with nGVS could be caused by the postural response of the movement strategies, the assessment of muscle activity and joint motion in the lower limbs is crucial to elucidate the effects of nGVS on the postural control system.

Simultaneous measurements of surface electromyography (EMG) and inertial measurement units (IMU) are capable of a detailed real-time evaluation of multiple joint movements and muscle contractions (Siebers et al., 2020). This measurement method has the advantage of evaluating physical function in various situations where movements are not limited by wearable devices and thus, can simultaneously measure muscle activity, joint movements, and body sway of the lower limbs. If physical control strategies during nGVS are evaluated for different conditions of the floor surface and visual information, it would provide valuable information, which can be used as a novel treatment method for balance dysfunction.

This study aimed to assess the changes in the lower limb muscle activity, joint movement, and body sway under different standing conditions with nGVS intervention using the simultaneous measurement method of surface EMG and IMU.

## Methods

### Participants

Seventeen healthy participants (mean age, 21.7 ± 2.3 years; six men) were included in this study. The participants had no history of neurological diseases, orthopedic diseases, or vestibular dysfunctions, such as vertigo, which could affect their standing posture control function. This study was approved by the Ethics Committee of Fukuoka International University of Health and Welfare (approval number 20-fiuhw-011) and was conducted in accordance with the principles and guidelines of the Declaration of Helsinki. All the participants provided written informed consent after the nature and purpose of the study were explained.

### Experimental Procedures

This study adopted a double-blind prospective design with blinding of the participants and evaluator. Participants performed in both the sham stimuli and nGVS interventions. The order of these interventions was randomized, and each intervention was conducted on a different day to avoid carryover effects. The evaluator measured the participants' data blindly. The other evaluators who were aware of the stimulus order were not involved in the recording or processing of the data.

**Figure 1:**
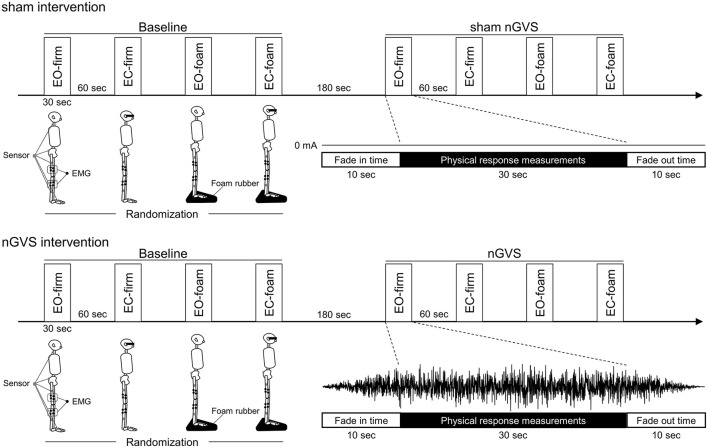
Experimental procedures. After measuring the physical responses in the standing posture in four different conditions (baseline), measured for the participants were performed in the same conditions during stimulation (sham or nGVS). The four conditions were randomized as follows: standing on a firm surface with eyes-open (EO-firm), standing on a firm surface with eyes-closed (EC-firm), standing on a soft foam with eyes-open (EO-foam), and standing on a soft foam with eyes-closed (EC-foam). Physical responses including the muscle activities of the rectus femoris, semitendinosus, tibialis anterior, and soleus muscles, and the angular velocity of the hip, knee, and ankle joints, as well as pelvic and neck body sway were measured in each condition.

The participants underwent physical response measurements using multiple surface EMGs and accelerometers under four conditions without stimulation (baseline) and four conditions with stimulation (sham or nGVS) (Figure 1). The four conditions were standing on a firm surface with eyes open (EO-firm), standing on a firm surface with eyes closed (EC-firm), standing on a soft foam with eyes open (EO-foam), and standing on a soft foam with eyes closed (EC-foam). The four conditions were performed in a random order. For all the conditions, the participants were asked to stand barefoot in an upright position with their feet together. For the eyes-open condition, the participants were asked to gaze at a target (1.0 cm diameter) 1.5 m away placed at their eye level. The participants were asked to stand quietly on a force plate. The foam rubber was made of a material with a tensile strength of 2.1 kgf/cm^2^ and thickness of 3.5 cm (Anima Co, Tokyo, Japan).

There were 60-s rest periods between the conditions, and 180-s rest periods between the baseline and stimulus (sham or nGVS) measurements. Noise stimulation (0–250 Hz) was applied to the nGVS session and sham stimulation (0 mA) was applied to the control session during the stimulation phase. Ramp up and ramp down times were both set to 10 s, and physical response measurements were performed during stimulation (30 s) following the ramp up time (Figure 1).

### nGVS

All nGVS was performed according to previous studies using DC-STIMULATOR PLUS (Eldith, NeuroConn GmbH, Ilmenau, Germany) (Inukai et al., 2018b; Helmchen et al., 2019; Matsugi et al., 2020a). Following skin preparation to reduce impedance (<10 kΩ), Ag/AgCl rubber electrodes (30 × 30 mm) were affixed to the left and right mastoid processes with random noise galvanic stimulation of the primary vestibular nerve. For nGVS, a random current level was generated for every sample (sampling rate: 1,280 samples/s) (Moliadze et al., 2012; Inukai et al., 2018b; Matsugi et al., 2020a). Statistically, the random numbers were normally distributed over time, the probability density followed a Gaussian bell curve, and all the coefficients featured a similar size in the frequency spectrum (Matsugi et al., 2020a). A waveform was applied with 99% of the values between −0.5 and +0.5 mA, and only 1% of the current level was within ±0.51 mA.

First, stimulus intensity tests were performed in participants in both the sham and nGVS conditions. The stimulus intensity test was initiated at 0.5 mA with a stimulus duration of a few seconds in a barefoot, upright, closed-eyed standing position with their feet together, similar to the physical response measurements. Thereafter, the intensity was gradually decreased in 0.025 mA increments until the participant reported no response to any skin perception, vestibular perception, or body movements. Perceptual thresholds were adjusted for the stimulus intensity until a stable response was obtained (Helmchen et al., 2019). Finally, the stimulus intensity was set at 80% of the perceptual threshold (Helmchen et al., 2019). If the participants perceived sham or nGVS, they were excluded from the study owing to lack of blinding. No participants were excluded from the present study.

### Physical Response Measurements

#### Muscle Activity

The surface EMG signal was captured using an 8-channel wireless EMG system (Clinical DTS, Noraxon USA Inc., AZ, USA) with a 16-bit resolution and common-mode rejection ratio >100 dB. Following proper skin preparation, eight circular Ag/AgCl surface electrodes (electrode diameter: 34 mm, inter-electrode distance: 30 mm) were placed, two each of the rectus femoris, semitendinosus, tibialis anterior, and soleus muscles of the right leg only under the assumption of symmetry, following the recommendations of the Surface EMG for Non-Invasive Assessment of Muscles (Hermens et al., 2000). The raw signals were pre-amplified 1,000 times and sampled at 1,500 Hz with a 500 Hz low-pass filter. After the postural stability test, the maximal voluntary contraction (MVC) of each muscle was obtained for 5 s using manual resistance (Murley et al., 2009; Huang et al., 2020), and each MVC test was repeated three times (Supplementary Material 1).

Left and right EMG activity and joint angular velocity were measured in eight healthy participants to confirm the symmetry of both the lower limbs. No significant differences were observed in any of the parameters (Supplementary Material 2).

#### Lower Limb Joint Motion

The participants' lower limb joint angular velocity was measured in the standing position using an IMU (Myomotion, Noraxon USA Inc., AZ, USA). Five sensors were placed based on the following anatomical landmarks: one on each foot, one on the right shank, one on the right thigh, and one on the center of the pelvis. Anatomic angles were calculated using the neutral-zero method. Based on the inertial sensor data, the joint angles in the three directions of the body segments (hip, knee, and ankle joints) were recorded, and the average values of these angular velocities were calculated by differentiating the joint angles obtained from each sensor (Struzik et al., 2015, 2016). All the measurements were recorded at a sampling frequency of 1,500 Hz.

#### Body Sway

The participants' body sway was measured in each standing position using the same IMU (Myomotion, Noraxon USA Inc., AZ, USA) that measured the lower limb joints' angular velocities. Two additional sensors were placed based on the following anatomical landmarks: the seventh cervical spinous process and the center of the pelvis (the same location as in the lower limb joints' angle measurements). Linear acceleration data from each sensor were recorded along the vertical, mediolateral, and anteroposterior directions. Considering the body sway, the root mean square (RMS) of each vertical, mediolateral, and anteroposterior direction were extracted and averaged to calculate the postural stability values as RMS sways. All the measurements were recorded at a sampling frequency of 1,500 Hz.

#### Data Extraction and Processing

The EMG and sensor data were processed using MyoResearch 3 (Noraxon USA Inc., AZ, USA). All the data were filtered using a first-order high-pass Butterworth filter at 10 Hz with an 8–10% cutoff. EMG data were then rectified, and the RMS (EMGrms) was calculated in 100 ms windows during the 30 s time window of the static standing trials. For each muscle, the highest EMGrms portion of 100 ms duration from the three MVC trials for each muscle was extracted and averaged and then used for normalization of the EMGrms value in each testing condition (Huang and Pang, 2019).

Given that the baseline values may differ between conditions, we calculated relative values by subtracting baseline data from intervention data for EMG activity, angular velocity, and RMS sway.

### Statistical Analysis

Three-way analysis of variance (ANOVAs) with repeated measure was conducted to determine the effects of measurement type, condition (EO-firm, EC-firm, EO-foam, and EC-foam), and intervention (sham and nGVS) on EMG activity, angular velocity, and RMS sway. Measurement type was defined as muscle type (rectus femoris, semitendinosus, tibialis anterior, and soleus muscles) for EMG activity, joint direction (hip flexion-extension, abduction-adduction, internal rotation-external rotation, knee flexion-extension, ankle dorsiflexion-plantar flexion, inversion-eversion, and abduction-adduction) for angular velocity, and body location (pelvis and neck) for RMS sway. EMG activity, angular velocity, and RMS sway may change between conditions due to differences in motor strategies regardless of intervention. To evaluate physical response modulated by condition, the baseline data were also compared using the three-way repeated measure ANOVA with effects of measurement type, condition, and intervention on EMG activity, angular velocity, and RMS sway. The effect sizes were evaluated according to the standardized size effect measure of partial eta squared ($\eta_{\text{p}}^{2}$). When an interaction was found within each parameter, a simple main effect analysis and a *post hoc* test with Bonferroni correction were conducted.

Statistical analyses were performed using IBM SPSS^®^ statistical software version 26 (IBM Corporation, Armonk, NY, USA). Statistical significance was set at *p* < 0.05.

## Results

Following randomization, eight participants received sham stimulation after nGVS, and nine participants received sham stimulation followed by nGVS. The average stimulus intensity of the nGVS in the present study was 0.37 ± 0.06 mA. The intervention interval between sham and nGVS was 11.1 ± 14.3 days. None of the participants reported any adverse events during or after the intervention. Baseline EMG activity, angular velocity, and RMS sway data are presented in Table 1. Each parameter during the intervention is presented in Supplementary Material 3.

**Table 1:** Baseline EMG activity, angular velocity, and root mean square sway in each condition.

	**Sham stimulation**				**nGVS**			
	**EO-firm**	**EC-firm**	**EO-foam**	**EC-foam**	**EO-firm**	**EC-firm**	**EO-foam**	**EC-foam**
**EMG activity (%)**								
Rectus femoris muscle	1.45 ± 1.77	1.40 ± 1.52	2.02 ± 2.25	2.57 ± 2.93	1.28 ± 1.48	1.65 ± 1.76	1.89 ± 1.52	2.76 ± 2.58
Semitendinosus muscle	0.95 ± 0.90	1.05 ± 1.18	1.52 ± 2.04	2.55 ± 2.84	1.05 ± 1.07	0.85 ± 0.62	1.26 ± 1.16	1.95 ± 1.26
Tibialis anterior muscle	1.01 ± 0.39	1.05 ± 0.63	1.85 ± 1.19	4.12 ± 2.77	1.08 ± 0.53	1.44 ± 1.15	2.48 ± 1.94	4.87 ± 3.17
Soleus muscle	7.11 ± 3.39	7.11 ± 3.20	9.93 ± 3.86	11.28 ± 3.85	8.43 ± 3.47	7.91 ± 3.91	9.06 ± 3.04	11.88 ± 5.42
**Angular velocity (deg/s)**
Hip flexion-extension	0.48 ± 0.19	0.80 ± 0.61	0.84 ± 0.63	1.17 ± 0.53	0.55 ± 0.26	0.53 ± 0.20	0.84 ± 0.45	1.20 ± 0.61
Hip abduction-adduction	0.29 ± 0.12	0.38 ± 0.22	0.71 ± 0.29	1.30 ± 0.62	0.31 ± 0.13	0.35 ± 0.11	0.78 ± 0.35	1.20 ± 0.54
Hip internal-external rotation	0.65 ± 0.44	0.84 ± 0.57	1.76 ± 1.14	2.78 ± 0.98	0.67 ± 0.32	0.88 ± 0.47	1.65 ± 0.79	2.83 ± 1.16
Knee flexion-extension	0.36 ± 0.20	0.65 ± 0.59	0.79 ± 0.49	1.23 ± 0.49	0.39 ± 0.24	0.42 ± 0.18	0.81 ± 0.50	1.27 ± 0.73
Ankle dorsiflexion-plantar flexion	0.38 ± 0.25	0.50 ± 0.23	1.24 ± 0.42	2.27 ± 0.66	0.91 ± 1.36	0.52 ± 0.36	1.37 ± 0.65	2.56 ± 1.33
Ankle inversion-eversion	0.64 ± 0.51	0.44 ± 0.25	1.51 ± 0.95	2.68 ± 2.44	0.86 ± 1.33	0.50 ± 0.38	1.67 ± 0.90	2.98 ± 1.95
Ankle abduction-adduction	0.64 ± 0.51	0.78 ± 0.54	1.65 ± 1.05	2.44 ± 1.06	0.77 ± 0.41	0.80 ± 0.42	1.54 ± 0.78	2.62 ± 1.20
**Root mean square sway**								
Pelvis	2.10 ± 0.80	2.06 ± 0.75	2.07 ± 0.77	1.93 ± 0.73	2.26 ± 0.86	2.18 ± 0.74	2.14 ± 0.74	2.18 ± 0.74
Neck	4.50 ± 1.20	4.45 ± 1.30	4.53 ± 1.20	4.65 ± 1.18	4.47 ± 1.31	4.54 ± 1.29	4.48 ± 1.11	4.70 ± 1.22

*The data are expressed as the means and the standard deviations. nGVS, noisy galvanic vestibular stimulation; EMG, electromyography; EO-firm, firm surface with eyes-open; EC-firm, firm surface with eyes-closed; EO-foam, soft foam with eyes-open; EC-foam, soft foam with eyes-closed*.

Regarding the baseline data, there were significant main effects of EMG activity (*F* = 296.509, *p* < 0.001, $\eta_{\text{p}}^{2}$ = 0.635), angular velocity (*F* = 33.423, *p* < 0.001, $\eta_{\text{p}}^{2}$ = 0.183), and RMS sway (*F* = 381.100, *p* < 0.001, $\eta_{\text{p}}^{2}$ = 0.598) in the measurement type, and EMG activity (*F* = 29.785, *p* < 0.001, $\eta_{\text{p}}^{2}$ = 0.149) and angular velocity (*F* = 225.288, *p* < 0.001, $\eta_{\text{p}}^{2}$ = 0.430) in the condition (Table 2). There was a significant interactive effect of EMG activity (*F* = 2.614, *p* = 0.006, $\eta_{\text{p}}^{2}$ = 0.044) and angular velocity (*F* = 7.134, *p* < 0.001, $\eta_{\text{p}}^{2}$ = 0.125) in the measurement type × condition (Table 2). *Post hoc* analysis showed that the EMG activity of the tibialis anterior, and soleus muscles was significantly greater in the EC-foam condition than that in the other conditions (tibialis anterior muscle: EO-firm, *p* < 0.001; EC-firm, *p* < 0.001; EO-foam, *p* < 0.001; soleus muscle: EO-firm, *p* = 0.014; EC-firm, *p* = 0.005) (Supplementary Material 4). The angular velocity in all the directions of motion was significantly greater in the EC-foam condition than that in the other conditions (*p*s < 0.05) (Supplementary Material 4).

**Table 2:** Results of repeated measures three-way ANOVA for baseline EMG activity, angular velocity, RMS sway.

	**EMG activity**			**Angular velocity**			**RMS sway**		
	***F***	***p***	** $\eta_{p}^{2}$ **	***F***	***p***	** $\eta_{p}^{2}$ **	***F***	***p***	** $\eta_{p}^{2}$ **
Measurement type	296.509	**<0.001**	0.635	33.423	**<0.001**	0.183	381.100	**<0.001**	0.598
Condition	29.785	**<0.001**	0.149	225.288	**<0.001**	0.430	0.047	0.987	0.001
Intervention	0.728	0.394	0.001	2.040	0.154	0.002	0.437	0.509	0.002
Measurement type × condition	2.614	**0.006**	0.044	7.134	**<0.001**	0.125	0.312	0.817	0.004
Measurement type × intervention	0.669	0.571	0.004	1.112	0.353	0.007	0.280	0.597	0.001
Condition × intervention	0.286	0.836	0.002	1.265	0.285	0.004	0.058	0.982	0.001
Measurement type × condition × intervention	0.414	0.928	0.007	0.214	1.000	0.004	0.021	0.996	0.000

*Measurement type was defined as muscle type for EMG activity, joint direction for angular velocity, and body location for RMS sway. ANOVA, analyses of variance; EMG, electromyography; RMS, root mean square. Bolded values indicate p < 0.05*.

**Figure 2:**
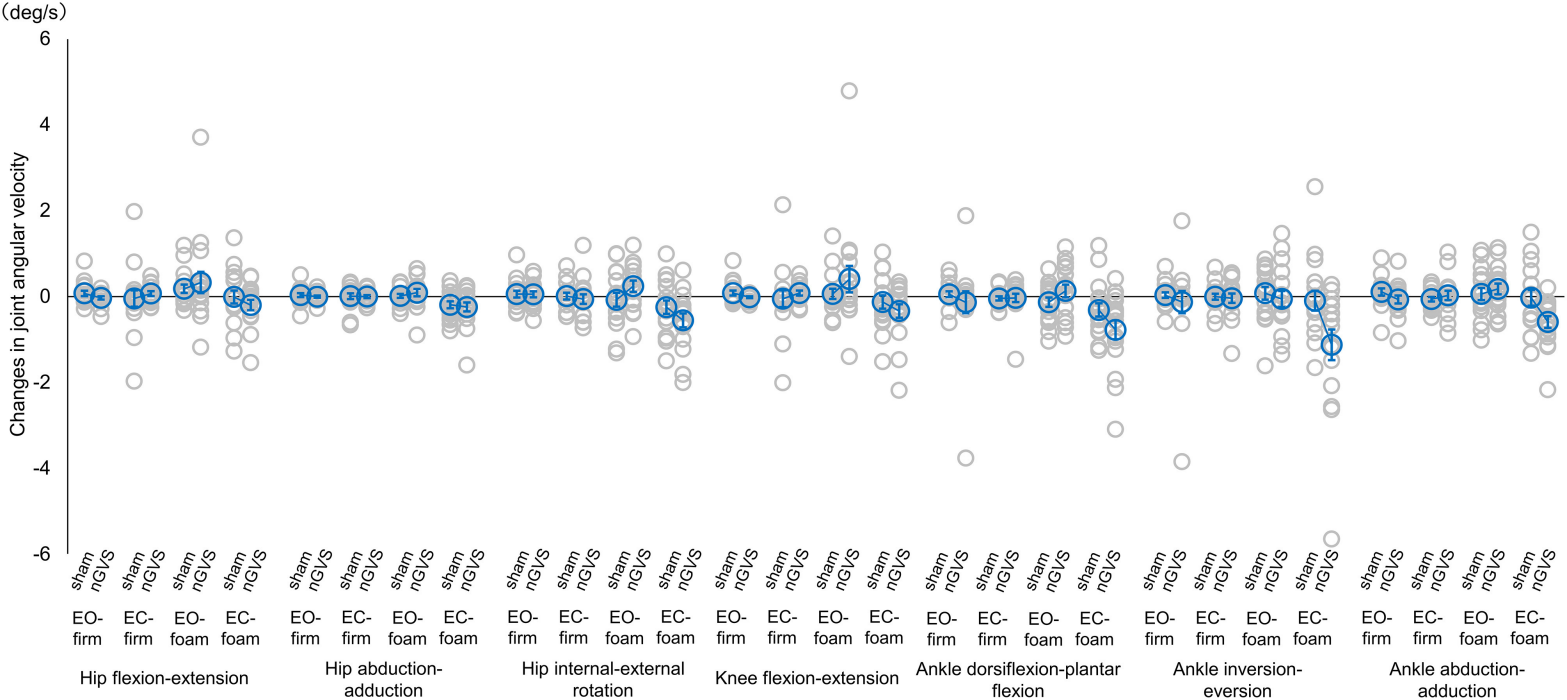
Mean joint angular velocity during sham or nGVS. Angular velocity was normalized by baseline activity. Accordingly, positive values indicate an increase in angular velocity during stimulation, while negative values indicate a decrease in angular velocity. Blue circles indicate mean joint angular velocity activity, and gray circles indicate joint angular velocity for each participant. Error bars indicate SEM. *p < 0.05. nGVS, noisy galvanic vestibular stimulation; EO-firm, firm surface with eyes-open; EC-firm, firm surface with eyes-closed; EO-foam, foam surface with eyes-open; EC-foam, foam surface with eyes-closed.

The main effects and interactions in the changes from the baseline for each parameter are presented in Table 3. There were significant main effects of angular velocity (*F* = 2.475, *p* = 0.022, $\eta_{\text{p}}^{2}$ = 0.016) in measurement type, EMG activity (*F* = 6.879, *p* < 0.001, $\eta_{\text{p}}^{2}$ = 0.039) and angular velocity (*F* = 26.698, *p* < 0.001, $\eta_{\text{p}}^{2}$ = 0.082) in condition, and EMG activity (*F* = 7.532, *p* = 0.006, $\eta_{\text{p}}^{2}$ = 0.014) and angular velocity (*F* = 4.338, *p* = 0.038, $\eta_{\text{p}}^{2}$ = 0.005) in intervention (Table 3). There were significant interactive effects of EMG activity (*F* = 3.684, *p* = 0.012, $\eta_{\text{p}}^{2}$ = 0.021) in measurement type × intervention, and EMG activity (*F* = 2.780, *p* = 0.041, $\eta_{\text{p}}^{2}$ = 0.016) and angular velocity (*F* = 10.052, *p* < 0.001, $\eta_{\text{p}}^{2}$ = 0.033) in condition × intervention (Table 3). For the angular velocity of each lower limb joint, the *post hoc* analysis revealed that the angular velocity was significantly decreased in the abduction-adduction direction in the EC-foam condition as compared to the EO-foam condition in the nGVS intervention (*p* = 0.005) (Figure 2). The ankle joint dorsiflexion-plantar flexion and inversion-eversion directions seemed to change significantly; however, there were no significant differences among the conditions or interventions. There were no significant differences in the interaction effects in RMS sway.

**Table 3:** Results of repeated measures three-way ANOVA for change in the EMG activity, angular velocity, and RMS sway.

	**EMG activity**			**Angular velocity**			**RMS sway**		
	***F***	***p***	** $\eta_{p}^{2}$ **	***F***	***p***	** $\eta_{p}^{2}$ **	***F***	***p***	** $\eta_{p}^{2}$ **
Measurement type	0.668	0.572	0.004	2.475	**0.022**	0.016	0.175	0.676	0.001
Condition	6.879	**<0.001**	0.039	26.698	**<0.001**	0.082	1.154	0.328	0.013
Intervention	7.532	**0.006**	0.014	4.338	**0.038**	0.005	1.258	0.263	0.005
Measurement type × condition	1.378	0.195	0.024	0.668	0.845	0.013	1.159	0.326	0.013
Measurement type × intervention	3.684	**0.012**	0.021	1.639	0.133	0.011	1.407	0.237	0.005
Condition × intervention	2.780	**0.041**	0.016	10.052	**<0.001**	0.033	0.416	0.742	0.005
Measurement type × condition × intervention	0.923	0.505	0.016	0.637	0.872	0.013	0.346	0.792	0.004

*Measurement type was defined as muscle type for EMG activity, joint direction for angular velocity, and body location for RMS sway. ANOVA, analyses of variance; EMG, electromyography; RMS, root mean square. Bolded values indicate p < 0.05*.

## Discussion

We investigated the effects of nGVS intervention on the lower limb muscle activity, joint motion, and body sway under different standing posture conditions. EMG activity and joint angular velocity had a significant effect at baseline, and we measured the change from baseline to intervention to observe the general intervention effects. This study found an interactive effect of the intervention with measurement type to EMG activity, and main effect of the intervention. Transcranial random noise stimulation, such as nGVS, synchronizes the firing of large populations of cells by propagation from a single cell to the neuronal population level, according to stochastic resonance mechanisms (Fröhlich and McCormick, 2010; Reato et al., 2010). This stimulation may modulate the neuronal activity of the primary vestibular afferents and vestibular hair cells (Gensberger et al., 2016; Herssens and McCrum, 2019). In addition, this stimulation effectively lowered the vestibular threshold involving balance-related reflexes, such as the vestibulospinal reflex, for appropriate postural adjustment (Schniepp et al., 2018). nGVS was effective in improving postural stability in healthy participants (Iwasaki et al., 2014). In the present study, the facilitation of balance-related reflexes by nGVS inhibited excessive body sway; therefore, the nGVS intervention could have changed the postural control responses demonstrated by the EMG activity.

On the other hand, a previous study demonstrated that nGVS with a stimulus intensity of 1 mA increased the EMG activity of the soleus muscle under EC-foam conditions (Matsugi et al., 2020b). Noise stimuli of appropriate intensity in various sensory functions detect established stochastic resonance as noise-enhanced responses of non-linear systems to weak signals (Richardson et al., 1998; Ries, 2007). Considering postural stability, Iwasaki et al. (2014) reported that low-intensity nGVS (≈0.28 mA) decreases postural sway. They proposed that an appropriate intensity of nGVS could be effective in detecting weak input signals *via* small changes in the transmembrane potentials (Iwasaki et al., 2014). Considering the interactive dependence of complex balance systems in a variety of environments (Horak, 2006), interventions on vestibular sensation, one of the sensory strategies, could influence the reactive postural control of motor strategies. Thus, it is possible that nGVS at appropriate stimulus intensities may have resulted in the facilitation of vestibular-related reflexes that detect detailed body movements, resulting in the change in physical activity. Future studies should investigate the changes in muscle activity and joint motion with different nGVS stimulation intensities.

A previous study showed that the center of pressure sway during EC-foam was decreased by the learning effect of repeating the same standing holding task (Inukai et al., 2020). However, the learning effect of repeated standing in the EC-foam did not extend to the decrease in the center of the pressure sway in the EO-firm (Inukai et al., 2020). The order of the four conditions in this study (EO-firm, EC-firm, EO-foam, and EC-foam) was randomized to avoid consecutively performing one condition. Therefore, this result suggests that the changes were attributed to the nGVS intervention and not to the effect of motor learning owing to repeated measurement of postural responses in each condition.

Considering the changes in the postural control responses between conditions owing to nGVS intervention, there were significant interactive effects of EMG activity and angular velocity in condition × intervention. The *post hoc* analysis revealed that the angular velocity was significantly decreased in the ankle joint abduction-adduction direction during the EC-foam condition compared with the EO-foam condition. Maintaining balance in the EC-foam requires the vestibular system to adapt to the changes in the visual and somatosensory inputs (Mitsutake et al., 2021). This study suggests that the nGVS intervention effectively reduces the compensatory strategy of the reactive postural control of the ankle joint motion in vestibular-dominant postural control conditions.

There was no significant difference in each joint angular velocity between sham and nGVS in the standing conditions. It is important to note that some participants showed increase in the angular velocity change with nGVS in the EO-foam condition. A previous study reported that dependence on visual, proprioceptive, or vestibular sensation in the quiet standing position was weighted differently by individual participants; thus, a certain number of participants showed increased body sway with visual stimulation compared to vestibular stimulation (Bonan et al., 2013). Participants with high visual dependence could have little benefit from nGVS in conditions requiring visual information, such as EO-foam. These participants might stabilize their posture by closing their eyes. In fact, this study demonstrated that the EC-foam condition significantly decreased the joint angular velocity with nGVS. However, the present study could not theoretically explain why some participants had increased angular velocity by nGVS in the EO-foam condition. The variability in sensory dependence could have affected the sensory re-weighting with nGVS; hence, postural stability could have been decreased by some sensory stimuli.

At baseline, there was an interaction between measurement type and condition in EMG activity and angular velocity, and the main effects of each parameter were also found. The *post hoc* analysis showed that the angular velocity tended to increase the parameters related to the hip motion in the EC-foam condition. Standing stabilization during the foam surface conditions is primarily achieved by an anti-phase relationship between the angular velocities of the upper and lower body through control at the hip joint (Fino et al., 2020). Considering the effect of hip strategy on postural control, the EC-foam condition possibly increased the periprosthetic hip muscle activity and joint motion. However, this study did not observe any difference in the EMG activity of the rectus femoris or semitendinosus muscles or in the angular velocity of the hip joint, even with stimulus intervention. Based on the closed-loop model of postural control, body sway is the result of an active corrective torque at the ankle proportional to the relative weighting of the visual, proprioceptive, and vestibular cues (Peterka, 2002). Thus, nGVS possibly provided postural adjustment focused on the ankle joint strategy and did not adapt to the postural control strategy of the hip joint.

There were no significant differences in the pelvic or neck RMS sway between the standing conditions in terms of the changes attributed to the stimulus intervention. A previous study found that high-frequency (100–500 Hz) transcranial random noise stimulation acutely increases the excitability of the cortical motor circuits, extending the principle of noise benefit to the neural population level of the human cortex (Potok et al., 2021). In contrast, another study showed that low-and high-frequency transcranial random noise stimulation might be modulated by different neural mechanisms (Saiote et al., 2013). The frequency of nGVS (0–250 Hz) used in this study could have affected the postural stability owing to the mixture of low (<100 Hz) and high (>100 Hz) frequencies. Similarly, the RMS sways of the pelvis and neck were not significantly different between the conditions, even at baseline. A previous study with participants whose average age was 37.7 ± 12.8 years showed significant differences in the body movements of the head and lumbar regions in the EC-foam condition compared to the EO-firm condition (Fino et al., 2020), which is inconsistent with the results of this study. The number of human vestibular ganglion neurons decreases with age (Park et al., 2001) and age-related changes in the EMG responses of the tibialis anterior and soleus muscles to the GVS (Welgampola and Colebatch, 2002; Dalton et al., 2014). This study performed the GVS on younger participants (21.7 ± 2.3 years) who could have adapted the smooth re-weighting of postural control, including vestibular perception and did not increase body sway even in the EC-foam condition owing to appropriate body control responses.

This study had several limitations. First, we measured the lower limb motion using only standardized angular velocity data. A previous study investigated the adaptation of the ankle and ankle-hip strategies based on the mean magnitude-squared coherence between the trunk-leg accelerations (Noamani et al., 2020). Another study measured the effect of co-contraction of the plantar and dorsiflexor muscles on the postural sway using a computational simulation study (Fok et al., 2021). Owing to the high accuracy of the instrumentation used in this study in capturing angular velocity at the ankle joint, future studies should include frequency and simulation analyses to investigate the effects of the ankle and hip strategies in more detail. Second, the EMG activity and IMU were evaluated by placing the device on only one lower limb because the trunk and head movements were simultaneously measured, and we needed to prioritize the comfort of the participants. It is crucial to simultaneously measure both the lower limbs to accurately capture the posture control response in future studies. Third, we did not clarify whether the decreased EMG activity and joint angular velocity were caused by nGVS-induced stochastic resonance. A previous study demonstrated that nGVS effectively reduces the vestibular motion perception thresholds in the presence of low-intensity stochastic vestibular stimuli (Galvan-Garza et al., 2018). However, another study reported that nGVS effects on body sway were incompatible with the stochastic resonance in healthy young adults (Assländer et al., 2021). Future studies should clarify whether the modulation of EMG activity and angular velocity is induced by the nGVS amplitude following stochastic resonance.

Despite these limitations, this study is the first to simultaneously measure nGVS-induced changes in muscle activity and joint motion in the lower limb. The results demonstrated that nGVS altered physical responses in different standing postural conditions. The present study is exploratory and therefore the evidence should be further investigated in future studies that specifically target those muscle activities and joint motion parameters.

## Data Availability Statement

The original contributions presented in the study are included in the article/Supplementary Material, further inquiries can be directed to the corresponding author/s.

## Ethics Statement

The studies involving human participants were reviewed and approved by Ethics Committee of Fukuoka International University of Health and Welfare. The patients/participants provided their written informed consent to participate in this study.

## Author Contributions

TM: conceptualization and writing—original draft. TM and HN: supervision and methodology. TM and TT: literature search and literature collection. TM, TT, HN, and HY: acquisition of data. TM, TT, and HN: interpretation of data. TM, TT, HN, HY, and MS: writing—review and editing. All authors contributed to the article and approved the submitted version.

## Funding

This work was supported by Takeda Science Foundation and by the Japan Society for the Promotion of Science (JSPS) KAKENHI Grant No. 20H04059.

## Conflict of Interest

The authors declare that the research was conducted in the absence of any commercial or financial relationships that could be construed as a potential conflict of interest.

## Publisher's Note

All claims expressed in this article are solely those of the authors and do not necessarily represent those of their affiliated organizations, or those of the publisher, the editors and the reviewers. Any product that may be evaluated in this article, or claim that may be made by its manufacturer, is not guaranteed or endorsed by the publisher.
