# Supplementary material for: Effects of Noisy Galvanic Vestibular Stimulation on the Muscle Activity and Joint Movements in Different Standing Postures Conditions
Source: Front Hum Neurosci. 2022 Jun 2;16:891669. doi: 10.3389/fnhum.2022.891669 (PMC9202802; doi:10.3389/fnhum.2022.891669)
Supplement: Supplementary file 1 [file Data_Sheet_1.PDF]

Supplementary Material

1 Supplementary File 1

Testing method for the maximum voluntary contractions (MVCs)

The MVC of each muscle was determined using a manual resistance test. To evaluate the MVC of the rectus femoris and semitendinosus muscles, each participant was seated on a chair with his/her thighs strapped together and knees flexed at 90°. Participants were asked to grasp the edge of the chair on each side for additional stabilization. The tested lower leg was strapped using a non-elastic belt attached to a fixed structure, and the participants were instructed to perform maximal isometric knee extension or flexion by pulling against the belt for five seconds. To test MVC during ankle plantar flexion and dorsiflexion, the knee was placed in a fully extended position with support, and the foot was placed in a neutral dorsiflexion/plantar-flexion position. To measure MVC during ankle plantar flexion, the ankle was in a neutral position, and the participants were instructed to isometrically push the ankle against the wedge with maximal effort for five seconds. To test ankle dorsiflexion, the participants were asked to perform maximal isometric ankle dorsiflexion by pushing against the researcher's hand for five seconds. Verbal encouragement in the form of standard scripts was provided during the muscle contractions. Three trials were conducted for each muscle.

2 Supplementary File 2

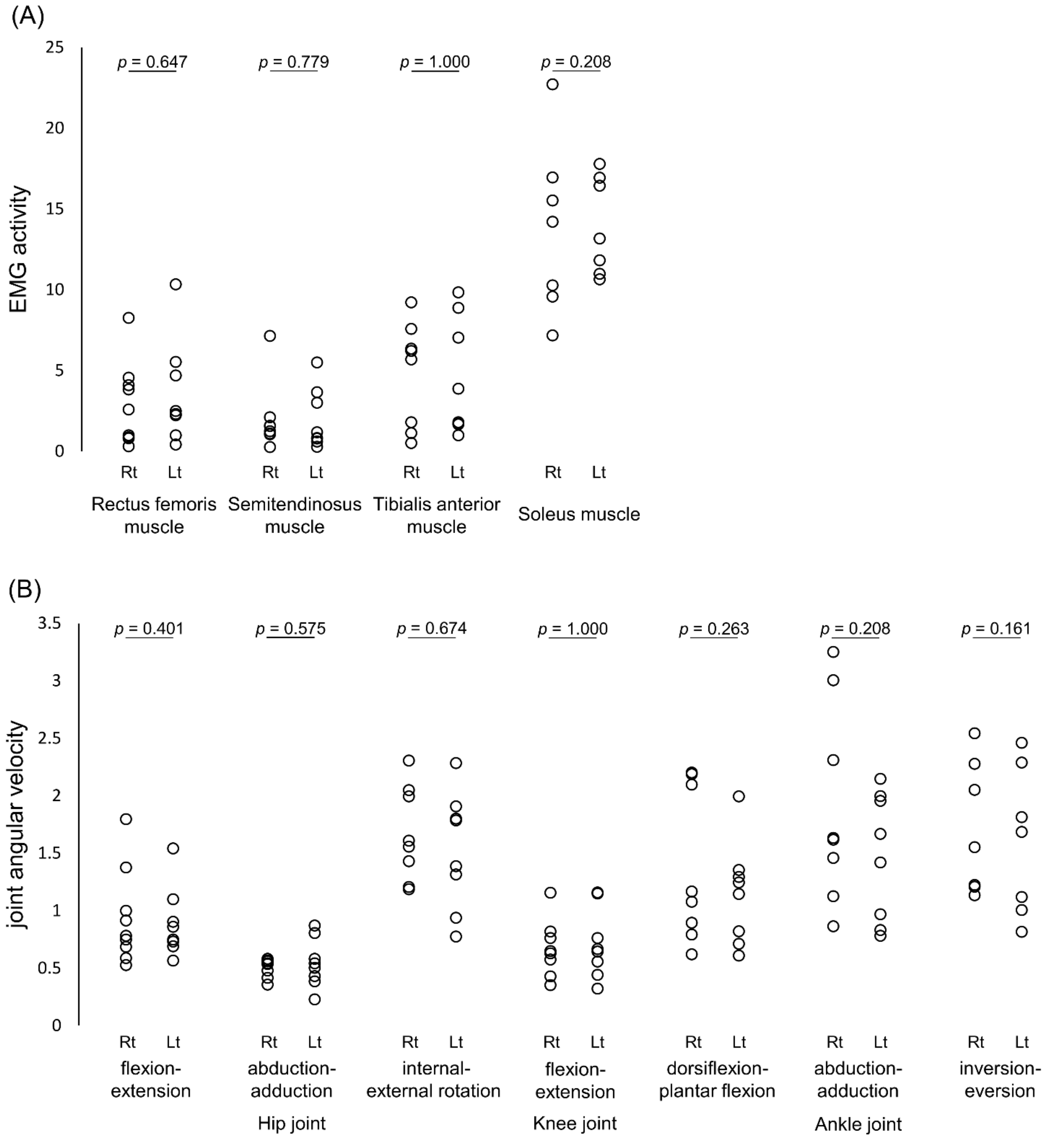

(A) Left-right comparison of EMG activity in lower limb muscles. (B) Left-right comparison of angular velocity for each joint in the lower limb.

Abbreviation: EMG, electromyography

3 Supplementary File 3

EMG activity, angular velocity, and root mean square sway during intervention in each condition.

	Sham stimulation				nGVS			
	EO-firm	EC-firm	EO-foam	EC-foam	EO-firm	EC-firm	EO-foam	EC-foam
EMG activity (%)								
Rectus femoris muscle	1.24 ± 1.53	1.39 ± 1.65	1.85 ± 2.05	2.50 ± 2.42	1.25 ± 1.98	1.46 ± 2.00	1.70 ± 1.38	2.02 ± 1.80
Semitendinosus muscle	0.88 ± 0.82	0.99 ± 1.11	1.10 ± 1.21	1.90 ± 1.79	0.86 ± 0.80	1.15 ± 1.15	0.94 ± 0.72	1.85 ± 2.20
Tibialis anterior muscle	1.23 ± 0.92	1.12 ± 0.62	1.77 ± 1.12	4.37 ± 2.91	1.29 ± 0.71	1.44 ± 0.98	2.13 ± 1.52	3.18 ± 1.75
Soleus muscle	7.26 ± 3.23	7.68 ± 3.02	10.07 ± 4.19	10.89 ± 4.27	7.93 ± 3.61	8.11 ± 3.58	8.30 ± 2.13	9.06 ± 2.55
Angular velocity (deg/s)								
Hip flexion-extension	0.54 ± 0.32	0.75 ± 0.71	1.01 ± 0.92	1.15 ± 0.68	0.51 ± 0.21	0.59 ± 0.29	1.16 ± 1.07	1.00 ± 0.42
Hip abduction-adduction	0.32 ± 0.19	0.38 ± 0.17	0.72 ± 0.25	1.10 ± 0.49	0.30 ± 0.14	0.34 ± 0.16	0.87 ± 0.42	0.95 ± 0.39
Hip internal-external rotation	0.70 ± 0.56	0.84 ± 0.53	1.68 ± 0.95	2.52 ± 1.15	0.71 ± 0.44	0.82 ± 0.47	1.88 ± 1.01	2.26 ± 1.27
Knee flexion-extension	0.44 ± 0.36	0.59 ± 0.69	0.85 ± 0.71	1.10 ± 0.66	0.37 ± 0.22	0.50 ± 0.34	1.21 ± 1.33	0.92 ± 0.47
Ankle dorsiflexion-plantar flexion	0.42 ± 0.26	0.45 ± 0.25	1.10 ± 0.44	1.95 ± 0.73	0.77 ± 1.55	0.48 ± 0.20	1.50 ± 0.81	1.78 ± 0.69
Ankle inversion-eversion	0.39 ± 0.31	0.43 ± 0.27	1.58 ± 0.93	2.58 ± 1.46	0.73 ± 1.48	0.46 ± 0.25	1.60 ± 0.97	1.85 ± 0.92
Ankle abduction-adduction	0.74 ± 0.49	0.71 ± 0.47	1.70 ± 0.97	2.40 ± 1.31	0.69 ± 0.49	0.82 ± 0.46	1.71 ± 1.02	2.02 ± 1.17
Root mean square sway								
Pelvis	2.14 ± 0.84	2.09 ± 0.78	2.07 ± 0.77	2.01 ± 0.79	2.26 ± 0.78	2.22 ± 0.86	2.24 ± 0.81	2.17 ± 0.76
Neck	4.41 ± 1.26	4.49 ± 1.33	4.45 ± 1.24	4.69 ± 1.23	4.48 ± 1.22	4.69 ± 1.27	4.46 ± 1.21	4.79 ± 1.24

The data are expressed as the means and the standard deviations. Abbreviation: nGVS, noisy galvanic vestibular stimulation; EMG, electromyography; EO-firm, firm surface with eyes-open; EC-firm, firm surface with eyes-closed; EO-foam, soft foam with eyes-open; EC-foam, soft foam with eyes-closed.

4 Supplementary File 4

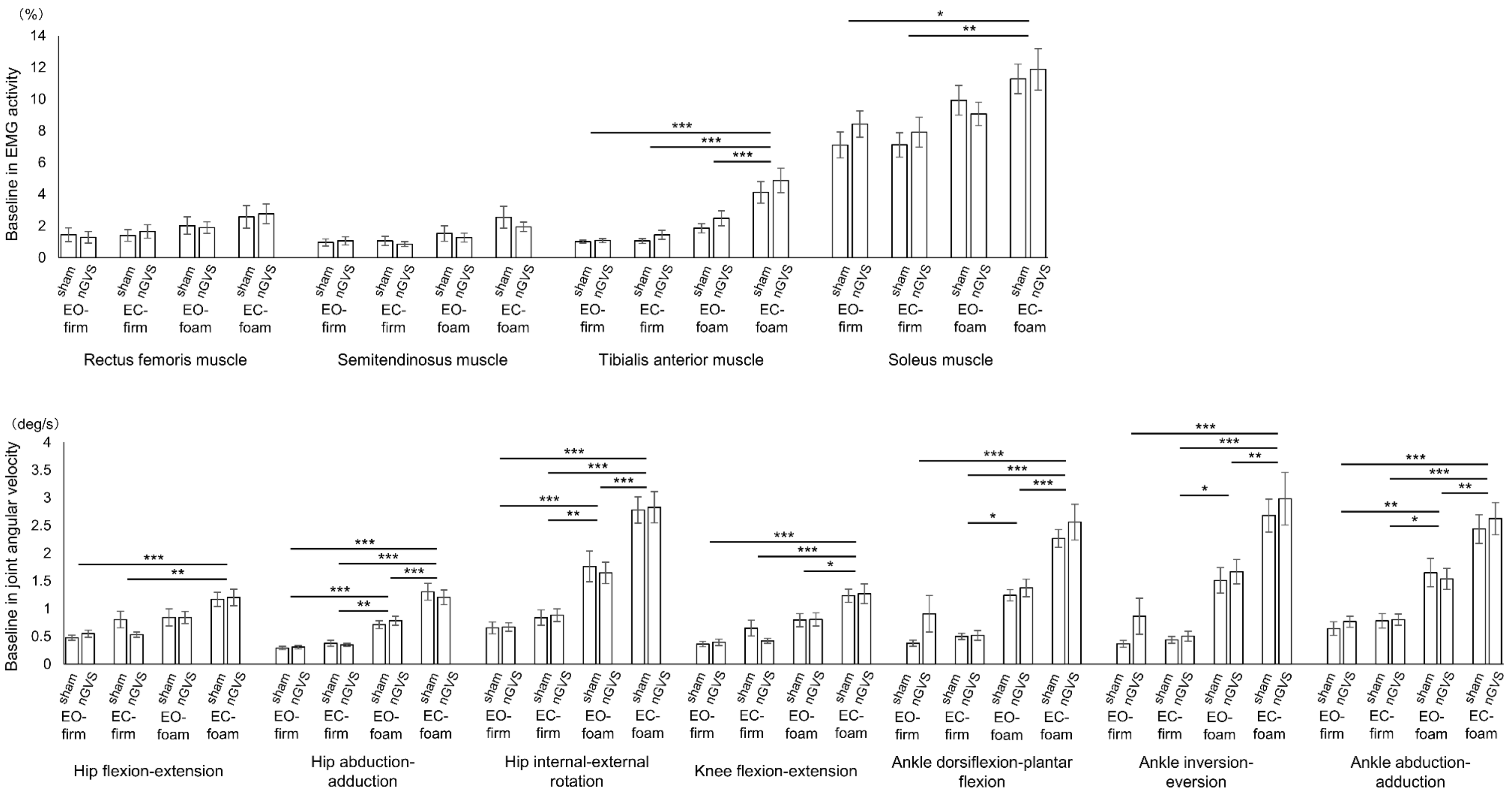

Baseline EMG activity, and angular velocity in each condition. Error bars indicate standard error of the mean (SEM). ****p* < .001; ***p* < 0.01; **p* < 0.05.

Abbreviation: EMG, electromyography; nGVS, noisy galvanic vestibular stimulation; EO-firm, firm surface with eyes-open; EC-firm, firm surface with eyes-closed; EO-foam, foam surface with eyes-open; EC-foam, foam surface with eyes-closed.
